# Prevention of Streptozotocin-Induced Diabetic Nephropathy by MG132: Possible Roles of Nrf2 and I*κ*B

**DOI:** 10.1155/2017/3671751

**Published:** 2017-03-08

**Authors:** Lili Kong, Yangwei Wang, Manyu Luo, Yi Tan, Wenpeng Cui, Lining Miao

**Affiliations:** ^1^Department of Nephrology, The Second Hospital of Jilin University, Changchun, Jilin, China; ^2^Department of Nephrology, The First Hospital of Jilin University, Changchun, Jilin, China; ^3^Department of Pediatrics, University of Louisville, Louisville, KY, USA

## Abstract

Our previous study showed that proteasomal inhibitor MG132 can prevent diabetic nephropathy (DN) along with upregulation of nuclear factor (erythroid-derived 2)-like 2 (Nrf2). The present study was to investigate whether MG132 can prevent DN in wild-type and Nrf2-KO mice. Type 1 diabetes was induced in wild-type and Nrf2-KO mice by multiple low doses of streptozotocin. Two weeks after streptozotocin injection, both wild-type and Nrf2-KO mice were randomly divided into four groups: control, MG132, DM, and DM/MG132. MG132 (10 *μ*g/kg/day) or vehicle was administered intraperitoneally for 4 months. Renal function, morphology, and biochemical changes were measured after 4-month treatment with MG132. MG132 treatment suppressed proteasomal activity in the two genotypes. In wild-type mice, MG132 attenuated diabetes-induced renal dysfunction, fibrosis, inflammation, and oxidative damage along with increased Nrf2 and I*κ*B expression. Deletion of* Nrf2* gene resulted in a partial, but significant attenuation of MG132 renal protection in Nrf2-KO mice compared with wild-type mice. MG132-increased I*κ*B expression was not different between wild-type and Nrf2-KO mice. This work indicates that MG132 inhibits diabetes-increased proteasomal activity, resulting in Nrf2 and I*κ*B upregulation and renal protection, which could be used as a strategy to prevent diabetic nephropathy.

## 1. Introduction

Diabetic nephropathy (DN) is the leading cause of end-stage renal failure worldwide. Additionally, DN is also linked to a high risk of cardiovascular disease. The risk factors include hyperglycemia, dyslipidemia, hypertension as well as elevation of homocysteine, and advanced glycation end products [1]. Moreover, albuminuria and glomerular filtration rate were also suggested to have relevant prognostic effects on cardiovascular morbidity and mortality, and the effect of albuminuria is especially pronounced when glomerular filtration rate is normal or near normal [2]. Both end-stage renal failure and cardiovascular disease brought us a heavy social burden. Current therapies for DN mainly including hypoglycemic agent and cotreatment with renoprotective drugs are not effective in blocking the progression of DN. Therefore, it is of vital importance and urgency to find more effective therapeutic strategies in countering the diabetes-associated renal injury.

Oxidative stress is induced by the imbalance of reactive oxygen species generation and endogenous antioxidant activity. Reactive oxygen species elicits inflammatory signaling pathways which in turn induces oxidative stress [3]. It is widely accepted that both oxidative stress and inflammation are main causes for DN [4, 5]. Thus, suppression of oxidative stress and inflammation may be an effective therapeutic strategy for DN.

The cell-permeable MG132 is a reversible, potent proteasome inhibitor. Reportedly, MG132 inhibited nuclear factor (erythroid-derived 2)-like 2 (Nrf2) and IkB proteasomal degradation, resulting in antioxidative stress and anti-inflammation function, respectively. Nrf2 is a transcription factor. By binding to the antioxidant-responsive element (ARE), Nrf2 could upregulate the expression of antioxidant genes and cytoprotective phase II detoxifying enzymes. Our previous study showed that nontoxic concentrations of MG132 could inhibit Nrf2 proteasomal degradation, leading to the renal protection of MG132 against diabetes-induced renal dysfunction [6]. Besides, MG132 was reported to inhibit IkB proteasomal degradation in myocardium [7]. Under physiological conditions, IkB binds to NF-*κ*B and retains NF-*κ*B in the cytoplasm, which prevents NF-*κ*B from activating the transcription of many inflammatory genes. MG132 upregulates IkB, resulting in transcriptional inactivation of NF-*κ*B and cardiac protection [7]. Therefore, MG132 may have the potential to treat DN through resisting oxidative stress and inflammation.

In the present study, we tried to address the question of whether proteasomal inhibitor MG132 can prevent diabetic nephropathy model in wild-type and Nrf2-KO mice induced by multiple low-dose streptozotocin. Also, we want to know whether renoprotection of MG132 was totally Nrf2-dependent.

## 2. Materials and Methods

### 2.1. Animals

Wild-type (Nrf2^+/+^), homozygote (Nrf2^−/−^), and heterozygote (Nrf2^+/-^) mice with C57BL/6J background were purchased from the Jackson Laboratory (Bar Harbor, Maine). Nrf2 knockout (KO, Nrf2^−/−^) male mice were obtained by breeding of heterozygote (Nrf2^+/−^) with homozygote (Nrf2^−/−^). Only wild-type and age-matched Nrf2 KO male mice were used for the present study. All experimental procedures for these mice were approved by the Institutional Animal Care and Use Committee of the University of Louisville, which is compliant with National Institutes of Health standards.

For induction of type 1 diabetic mouse model, 8-week-old male wild-type and Nrf2-KO mice were injected with multiple low-dose streptozotocin (Sigma-Aldrich, St. Louis, MO, USA) intraperitoneally, dissolved in 0.1 M sodium citrate buffer (pH = 4.5) at 50 mg/kg body weight daily for 5 consecutive days, while age-matched control mice received multiple injections of the same sodium citrate buffer. Five days after the last injection, mice with hyperglycemia (blood glucose levels ≥ 250 mg/dL) were defined as diabetes mellitus (DM) as before [8]. Both wild-type and Nrf2-KO mice were randomly allocated into four groups (*n* = 7 at least per group): control, MG132, DM, and DM/MG132. Dose of MG132 was used based on our previous study [6]. MG132 (Sigma-Aldrich, St. Louis, MO) was dissolved in dimethyl sulfoxide at a concentration of 0.0025 *μ*g/mL and diluted with saline for injection. Both nondiabetic and diabetic mice further received subcutaneous injection of MG132 at 10 *μ*g/kg or vehicle daily for 4 months. At the end of the 4 months, the mice were euthanized, and their kidneys were harvested for analysis.

### 2.2. Mouse Urinary Albumin to Creatinine Ratio (UACR) Detection

Urinary albumin and urinary creatinine were measured according to manufacturers' procedures provided with these kits (Bethyl Laboratories Inc., Montgomery, TX; BioAssay Systems, Hayward, CA, resp.). Mouse UACR was calculated as UACR = urinary albumin/urinary creatinine (*μ*g/mg).

### 2.3. Renal Histopathological Examination

Kidney tissues were fixed immediately in 10% buffered formalin solution after harvesting and were embedded in paraffin and sectioned into 5 *μ*m-thick sections onto glass slides. The sections were processed for PAS and Masson's trichrome staining.

### 2.4. Isolation of Nuclei

The nuclei from kidney tissue were isolated according to manufacturers' procedures provided with the nuclei isolation kit (Sigma-Aldrich). Renal tissue from each mouse was homogenized in cold lysis buffer containing dithiothreitol (DTT) and Triton X-100. Then, Cushing solution (sucrose Cushion solution : sucrose Cushion buffer : dithiothreitol = 900 : 100 : 1) was added and the mixture was transferred to a new tube preloaded with sucrose Cushion solution followed by centrifugation at 13,000 rpm for 45 min. The supernatant fraction containing cytosolic components was aspirated and the nuclei were visible as a thin pellet at the bottom of the tube.

### 2.5. Real-Time PCR

Real-time PCR were performed as previously described [9] using primers for NQO-1, Nrf2, and actin (Life Technologies, Grand Island, NY).

### 2.6. Western Blotting Assay

Western blotting assay was conducted as previously described [10]. The primary antibodies were FN (1 : 200 dilution), TGF-*β* (1 : 1000 dilution), 3-NT (1 : 1000 dilution), 4-HNE (1 : 1000 dilution), IL-6 (1 : 500 dilution), NF-*κ*B (1 : 1000 dilution), I*κ*B-*α* (1 : 1000 dilution), Nrf2 (1 : 500 dilution), actin (1 : 3000 dilution), and *α*-tubulin (1 : 2000 dilution), all of which were purchased from Santa Cruz Biotechnology except for 3-NT (Millipore), 4-HNE (Alpha Diagnostic), and TGF-*β*, NF-*κ*B, I*κ*B-*α*, and *α*-tubulin (Cell Signaling).

### 2.7. 20S Proteasome Activity Assay

The 20S proteasome, the catalytic core of the 26S proteasome complex, is responsible for the degradation of short-lived regulatory proteins, including Nrf2 and I*κ*B [7, 11, 12]. Since MG132 mainly inhibits proteasome chymotrypsin (ChT)-like activity [13], we detected 20S proteasome activity by quantifying the hydrolysis of SLLVY-AMC, a fluorogenic substrate for the ChT-like activity, according to the manufacturers' procedures of the 20S proteasome activity assay kit (Millipore). Detailed operation procedures have been described in our early studies [6].

### 2.8. Morphometric Analyses

Morphometric analyses were conducted using Image-Pro Plus 6.0 software (Media Cybernetics, Bethesda, MD, USA). Areas to be photographed were selected randomly by people blind to the identity of the samples.

### 2.9. Statistical Analysis

Data were collected from at least 7 mice each group and presented as means ± SD. Image Quant 5.2 was used to analyze western blotting. Comparisons among different groups were conducted by one-way ANOVA, followed by Tukey's post hoc test. In addition, a *t*-test was performed to compare the amount of decrease by MG132 between wild-type and Nrf2-KO mice. Differences were significant if *p* < 0.05.

## 3. Results

### 3.1. General Changes after STZ Injection for 5 Consecutive Days

After STZ injection for 5 consecutive days, diabetic mice developed hyperglycemia. There was no significance in UACR and body weight among the four groups (Table 1).

### 3.2. MG132 Retained Partial Protection against Diabetes-Induced Albuminuria despite Deletion of the Nrf2 Gene

**Figure 1:**
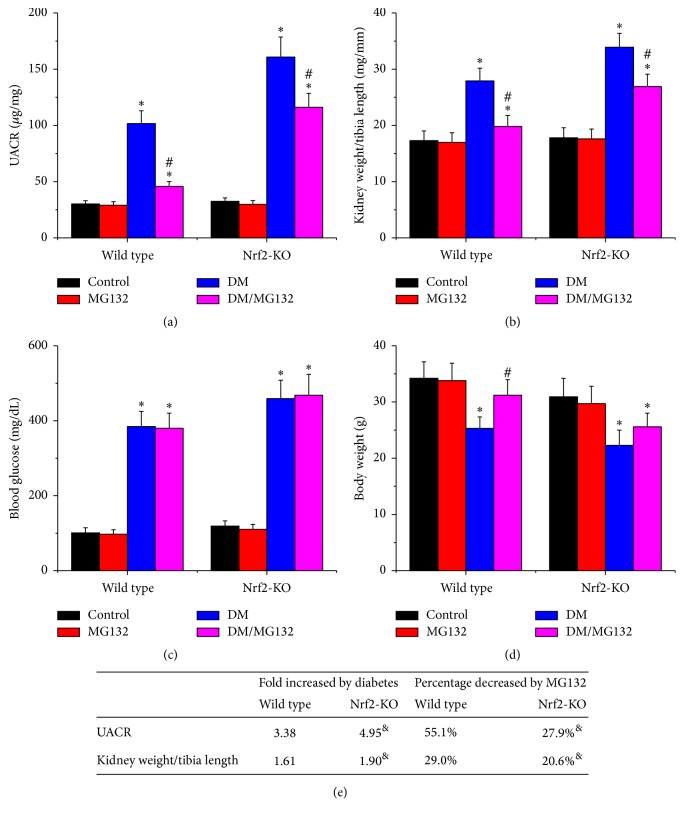
MG132 retained partial protection against diabetes-induced albuminuria despite deletion of the Nrf2 gene. UACR (a), kidney weight/tibia length (b), blood glucose (c), and body weight (d) were determined in all mice. Diabetes-induced pathological changes (fold) between wild-type and Nrf2-KO mice and the decreased percentages of these pathological changes with MG132 between WT and Nrf2-KO diabetic mice were compared (e). Data are presented as mean ± SD. ^*∗*^*p* < 0.05 versus WT/control or Nrf2-KO/control correspondingly; ^#^*p* < 0.05 versus WT/DM or Nrf2-KO/DM correspondingly; ^&^*p* < 0.05 versus wild-type mice.

As an important index of renal function, UACR was measured at the end of the study. As shown in Figures 1(a) and 1(e), compared to their respective controls, a 4.95-fold increase in UACR for Nrf2-KO diabetic mice and a 3.38-fold increase in UACR for wild-type diabetic mice were found. The results revealed that streptozotocin-injected Nrf2-KO mice had a higher level of UACR than wild-type mice, indicating the essential role of Nrf2 in protecting against streptozotocin-induced renal injury. Next, kidney weight/tibia length (Figures 1(b) and 1(e)), which indicates enlargement of kidney, was calculated. The ratio was significantly increased in the diabetic groups in both strains but was decreased by MG132 treatment. MG132 decreased UACR and kidney weight/tibia length by 55.1% and 29.0% in wild-type diabetic mice and by 27.9% and 20.6% in Nrf2-KO mice, respectively; these effects were significantly lower in Nrf2-KO mice (Figure 1(e)). It not only confirmed the pivotal role of Nrf2 in MG132 protection, but also proved an Nrf2-independent protection against diabetes-induced renal injury. Blood glucose (Figure 1(c)) was increased in both wild-type and Nrf2-KO diabetic mice, and MG132 had no significant impact on blood glucose in the two genotypes. Diabetes reduced body weight in both wild-type and Nrf2-KO diabetic mice (Figure 1(d)). Interestingly, MG132 increased body weight in wild-type diabetic mice, but not in Nrf2-KO diabetic mice.

### 3.3. MG132 Retained Partial Protection against Diabetes-Induced Renal Fibrosis despite Deletion of the Nrf2 Gene

**Figure 2:**
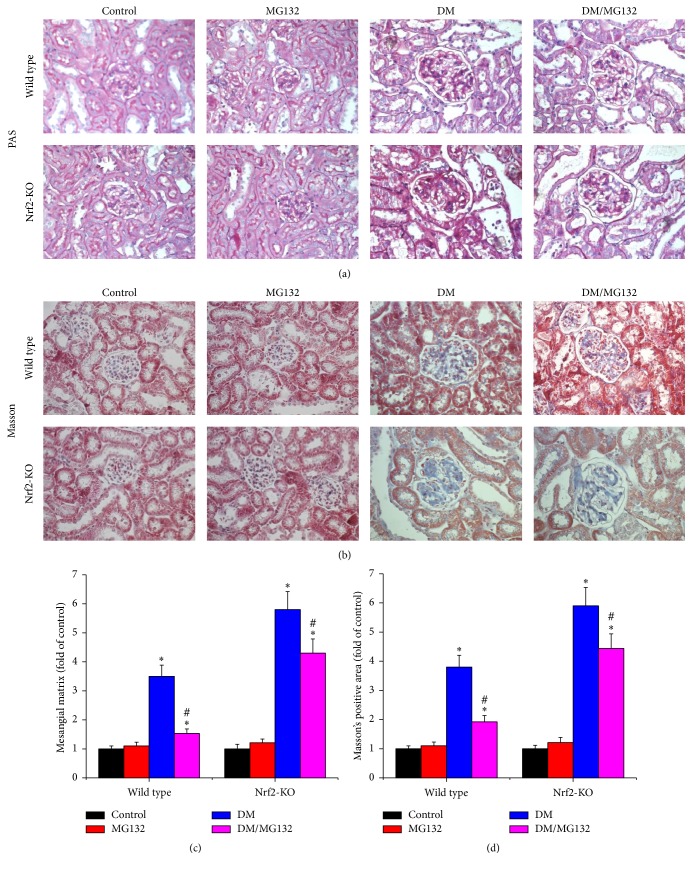
Effects of MG132 on diabetes-induced morphological changes were examined with PAS (a) and Masson's trichrome staining (b, ×400) in all mice. Mesangial matrix expansion (c) was quantified from PAS staining and fibrosis accumulation (d) was quantified from Masson's trichrome staining. Data are presented as mean ± SD. ^*∗*^*p* < 0.05 versus WT/control or Nrf2-KO/control correspondingly; ^#^*p* < 0.05 versus WT/DM or Nrf2-KO/DM correspondingly.

To investigate the effect of MG132 on diabetes-induced renal fibrosis, PAS staining (Figure 2(a)) was conducted to detect glycogen deposition and Masson's trichrome staining was conducted to measure the expression of fibronectin (FN) and collagens (Figure 2(b)). Diabetic kidney showed enlarged glomeruli, mesangial matrix expansion, and increased trichrome-positive area. MG132 significantly attenuated these changes in wild-type mice and still provided partial protection against diabetes-induced morphological changes in Nrf2-KO mice. Mesangial matrix expansion (Figure 2(c)) was quantified from PAS staining and fibrosis accumulation (Figure 2(d)) was quantified from Masson's trichrome staining.

**Figure 3:**
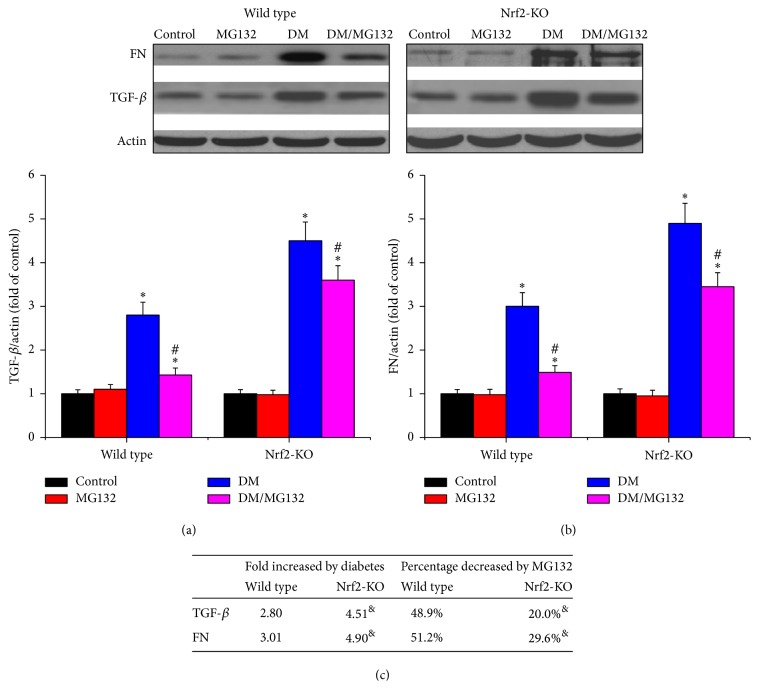
Effects of MG132 on diabetes-induced renal fibrosis in wild-type mice (a) and Nrf2-KO mice (b) were determined by detecting the expression of FN and TGF-*β* with western blotting assay. Diabetes-induced fibrotic changes (fold) between wild-type and Nrf2-KO mice and the decreased percentages of these changes with MG132 between WT and Nrf2-KO diabetic mice were compared (c). Data are presented as mean ± SD. ^*∗*^*p* < 0.05 versus WT/control or Nrf2-KO/control correspondingly; ^#^*p* < 0.05 versus WT/DM or Nrf2-KO/DM correspondingly; ^&^*p* < 0.05 versus WT mice.

Both FN and TGF-*β*, the two fibrosis indexes, were measured by western blotting assay in total proteins. The two kinds of protein were significantly increased in diabetic kidneys in the two genotypes, yet they were reduced by MG132 treatment. MG132 decreased FN (Figures 3(b) and 3(c)) and TGF-*β* (Figures 3(a) and 3(c)) by 51.2% and 48.9% in wild-type diabetic mice and by 29.6% and 20.0% in Nrf2-KO mice, respectively; these effects were significantly lower in Nrf2-KO mice.

### 3.4. MG132 Alleviated Diabetes-Induced Oxidative Stress in Wild-Type Diabetic Mice, but This Effect Was Completely Lost in Nrf2-KO Diabetic Mice

**Figure 4:**
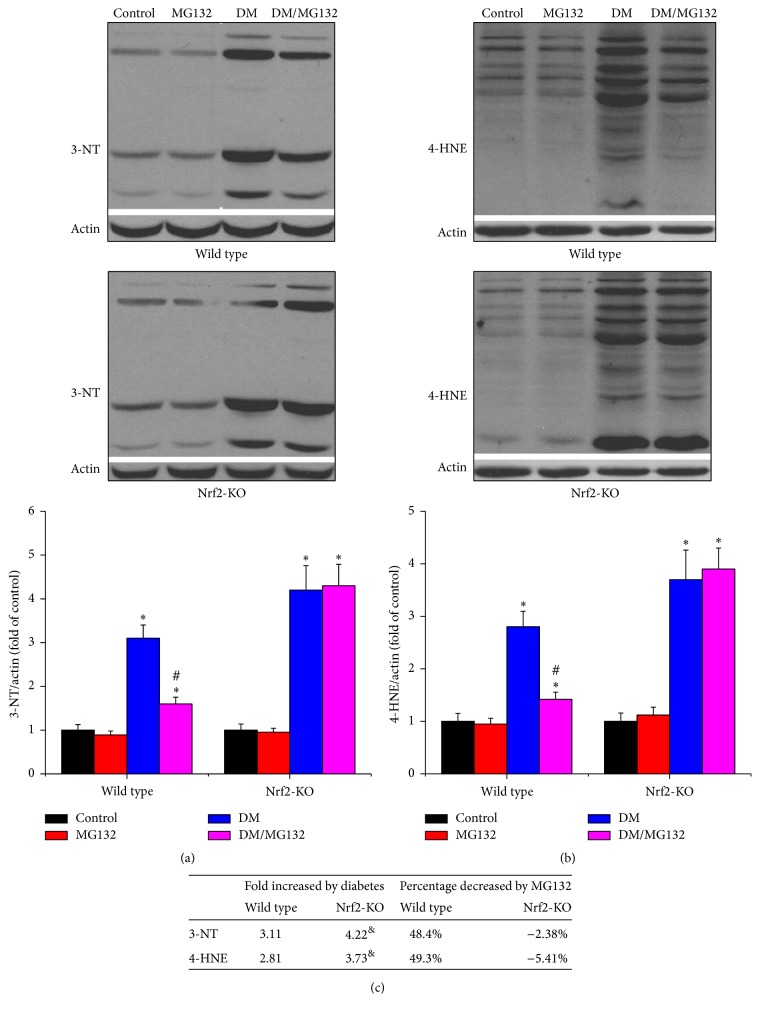
Effects of MG132 on diabetes-induced renal oxidative damage were determined by detecting the expression of 3-NT (a) and 4-HNE (b) with western blotting assay. Diabetes-induced changes of oxidative damage (fold) between wild-type and Nrf2-KO mice and the decreased percentages of these changes with MG132 between WT and Nrf2-KO diabetic mice were compared (c). Data are presented as mean ± SD. ^*∗*^*p* < 0.05 versus WT/control or Nrf2-KO/control correspondingly; ^#^*p* < 0.05 versus WT/DM or Nrf2-KO/DM correspondingly; ^&^*p* < 0.05 versus WT mice.

As shown in Figure 4, diabetes-induced oxidative damage was determined by 3-NT as an index of nitrosative damage and 4-HNE as an index of lipid peroxidation with western blotting assay in total proteins. Both 3-NT (Figures 4(a) and 4(c)) and 4-HNE (Figures 4(b) and 4(c)) were increased in diabetic kidney in the two genotypes, which was more obvious in Nrf2-KO mice. MG132 treatment significantly reduced 3-NT and 4-HNE accumulation in wild-type diabetic mice, but not in Nrf2-KO diabetic mice.

### 3.5. MG132 Retained Partial Protection against Diabetes-Induced Renal Inflammation despite Deletion of the Nrf2 Gene

**Figure 5:**
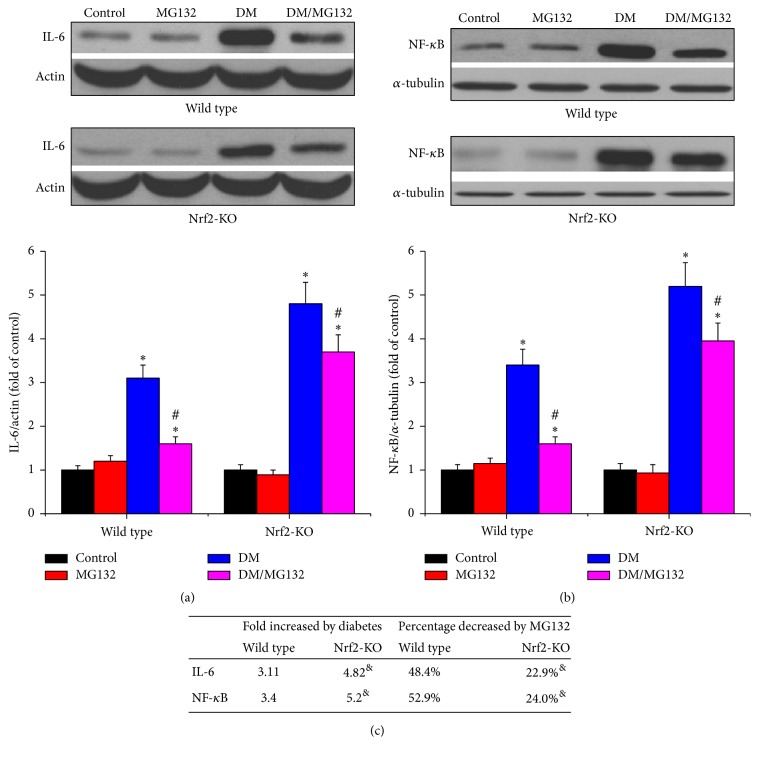
Effects of MG132 on diabetes-induced renal inflammation were determined by detecting the expression of IL-6 (a) and NF-kB (b) with western blotting assay. Diabetes-induced inflammatory changes (fold) between wild-type and Nrf2-KO mice and the decreased percentages of these changes with MG132 between WT and Nrf2-KO diabetic mice were compared (c). Data are presented as mean ± SD. ^*∗*^*p* < 0.05 versus WT/control or Nrf2-KO/control correspondingly; ^#^*p* < 0.05 versus WT/DM or Nrf2-KO/DM correspondingly; ^&^*p* < 0.05 versus WT mice.

As an important index of renal inflammation, IL-6 was determined by western blotting assay in total proteins, while NF-*κ*B was determined by western blotting assay in nuclear proteins. As shown in Figure 5, in both wild-type and Nrf2-KO mice, diabetes increased the expression of IL-6 (Figures 5(a) and 5(c)) and NF-*κ*B (Figures 5(b) and 5(c)) compared to control group, respectively. What is more, Nrf2-KO diabetic kidney expressed higher levels of IL-6 and NF-*κ*B, compared to wild-type diabetic kidney. MG132 decreased IL-6 and NF-*κ*B by 48.2% and 52.9% in wild-type diabetic mice and by 22.9% and 24.0% in Nrf2-KO mice, respectively; these effects were significantly lower in Nrf2-KO mice.

### 3.6. Possible Mechanisms by Which MG132 Attenuates DN

**Figure 6:**
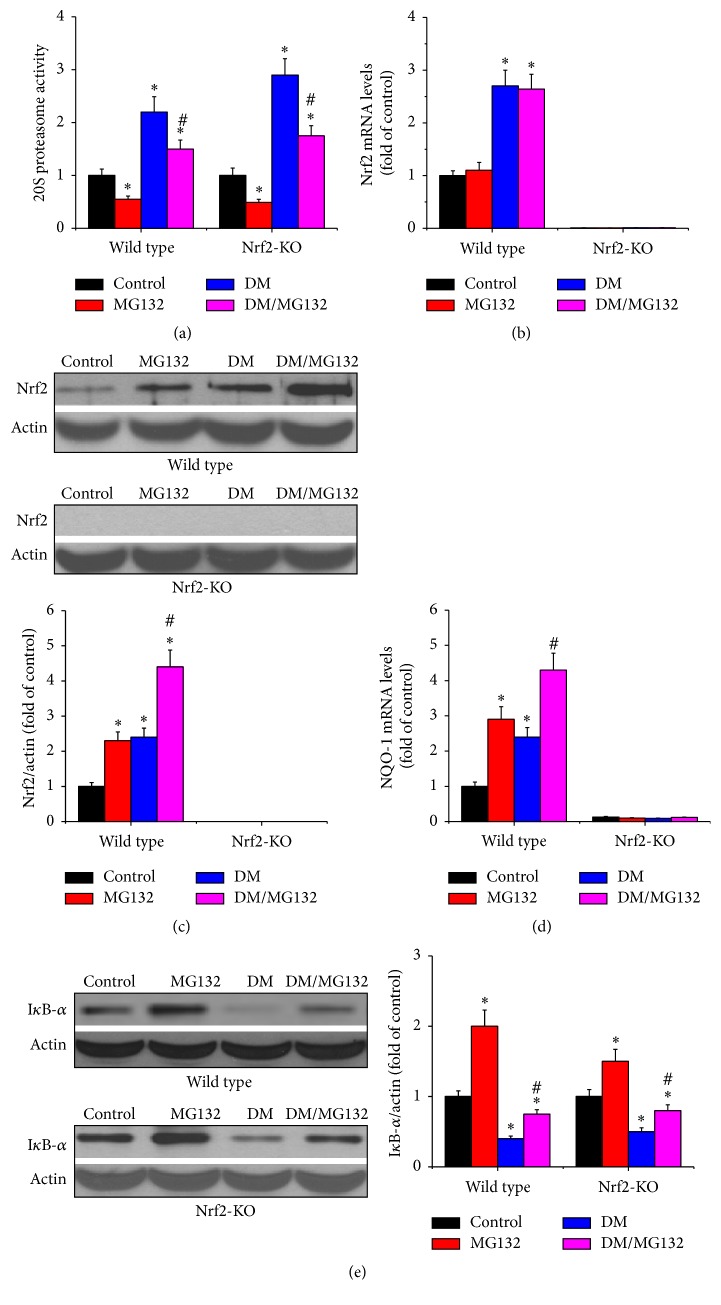
Possible mechanisms under which MG132 attenuates DN. 20S proteasome activity (a), Nrf2 expression at both mRNA (b) and protein levels (c), and Nrf2 downstream gene, NQO-1 mRNA (d) were examined in all mice. In addition, IkB-*α* protein level (e) was determined by western blotting assay. Data are presented as mean ± SD. ^*∗*^*p* < 0.05 versus WT/control or Nrf2-KO/control correspondingly; ^#^*p* < 0.05 versus WT/DM or Nrf2-KO/DM correspondingly.

Diabetes increased renal proteasomal activity, which was reduced by MG132. As shown in Figure 6(a), compared to respective control group, renal proteasomal activity was increased in diabetic group in the two genotypes and was significantly reduced by MG132 treatment.

### 3.7. MG132 Inhibited Renal Proteasomal Activity, Resulting in Upregulation of Nrf2

Ubiquitination and subsequent degradation by the proteasome have been regarded as the main mechanism responsible for Nrf2's negative regulation. MG132 inhibited proteasomal activity, which may result in the reduction of Nrf2 degradation. Therefore, Nrf2 mRNA (Figure 6(b)) and total protein levels (Figure 6(c)) were determined by real-time PCR and western blotting assay, respectively. Besides, NQO-1 expression, one of Nrf2 downstream genes, was also determined by real-time PCR (Figure 6(d)). In wild-type mice, diabetes increased Nrf2 expression at both mRNA and protein levels; MG132 treatment increased Nrf2 protein level, but not mRNA level. In Nrf2-KO mice, Nrf2 was almost undetectable by real-time PCR and western blotting assay. Consistent with Nrf2 protein levels, in wild-type mice, both diabetes and MG132 increased NQO-1 mRNA levels. However, Nrf2 deficiency disenabled MG132 to induce NQO-1 transcription.

### 3.8. MG132 Inhibited Renal Proteasomal Activity, Resulting in Upregulation of I*κ*B and Downregulation of NF-*κ*B

As a transcription factor, NF-*κ*B can translocate into the nucleus and transcriptionally upregulate inflammatory cytokines. I*κ*B is its negative regulator. Under basal conditions, I*κ*B binds to NF-*κ*B and retains NF-*κ*B in the cytoplasm, which reduced the transcriptional activity of NF-*κ*B. In order to determine the effect of MG132 on I*κ*B, we detected its protein level by western blotting assay in total proteins. As shown in Figure 6(e), in both wild-type and Nrf2-KO mice, diabetes significantly reduced the expressions of I*κ*B, which were significantly upregulated by MG132 treatment. This suggests that MG132 inhibited renal proteasomal activity, resulting in the reduction of I*κ*B degradation. Consequently, MG132 increased I*κ*B (Figure 6(e)) and reduced NF-*κ*B (Figure 5(b)).

## 4. Discussion

The present study is the first to demonstrate that MG132 attenuates DN via suppression of proteasomal activity of diabetic kidney, which promotes degradation of Nrf2 and I*κ*B. We set up diabetic mouse model with multiple low-dose streptozotocin in both wild-type and Nrf2-KO mice and treated with MG132 for 4 months. In wild-type mice, MG132 inhibited proteasomal activity, resulting in the significant upregulation of Nrf2 and I*κ*B. Consequently, MG132 significantly attenuated diabetes-induced renal dysfunction, fibrosis, inflammation, and oxidative damage. In Nrf2-KO mice, MG132 also inhibited proteasomal activity, resulting in the significant upregulation of I*κ*B. However, Nrf2 deficiency resulted in partial loss of MG132 protection against DN.

DN is characterized by inflammation, oxidative stress, enlarged glomeruli, expansion of mesangial matrix, glomerular basement membrane thickening, glomerulosclerosis, and tubulointerstitial fibrosis. Inflammation and oxidative stress are considered as main pathogenesis of DN. Increasing evidence indicated that blocking oxidative stress could attenuate diabetic complications, such as DN [14], diabetic retinopathy [15], and diabetic cardiomyopathy [16]. As a transcription factor, Nrf2 is a master regulator of cellular redox status. Under unstressed conditions, Nrf2 is kept in the cytoplasm by Kelch-like-ECH-associated protein 1 (Keap1) and Cullin 3 which induces ubiquitination of Nrf2 [17]. Once Nrf2 is ubiquitinated, it is transported to the proteasome, where it is degraded and its components are recycled. Under oxidative conditions, oxidative stress disrupts critical cysteine residues in Keap1, disrupting the Keap1-Cul3 ubiquitination system. Nrf2 is free from Keap1 and translocates from cytoplasm into the nucleus. In the nucleus, it combines with a small Maf protein and binds to the ARE in the upstream promoter region of genes encoding antioxidant enzymes and initiates their transcription [18]. Emerging evidence showed that upregulation of Nrf2 could alleviate oxidative injury and diabetic complications [19, 20]. Nrf2 is degraded by proteasome [21–24]; thus proteasomal inhibition might be a potent approach to upregulate Nrf2. Since the approval of the first proteasome inhibitor by the FDA [25], proteasome inhibitors have been used to treat several diseases. Due to the side effects and drug resistance, a new proteasome inhibitor without those disadvantages, namely, MG132, was discovered. It is reported that nontoxic concentrations of MG132 reduced Nrf2 proteasomal degradation, resulting in the upregulation of Nrf2 and its downstream antioxidants [26, 27]. Our previous study showed that MG132 prevents the development of DN in OVE26 mice via upregulation of Nrf2. Furthermore, silencing Nrf2 gene with siRNA disenabled MG132 to prevent renal tubule cells from high-glucose-induced profibrotic response. It suggested that therapeutic effect of MG132 on DN may be Nrf2-dependent. This present study also indicated the important role of Nrf2 in MG132 protection from DN. MG132, the proteasome inhibitor, upregulated Nrf2 and its downstream antioxidant genes, such as NQO-1, resulting in the alleviation of renal oxidative damage. In wild-type diabetic mice, MG132 reduced UACR by 55.1%. However, MG132 only reduced UACR in Nrf2-KO diabetic mice by 29.0%, demonstrating the beneficial effect of oxidative stress status alleviation on the renal protection afforded by MG132. Interestingly, MG132 retained partial protection against diabetes-induced renal injury in Nrf2-KO mice. That is, the renal protection of MG132 might not be Nrf2-dependent. There may be another mechanism underlying the protective effect of MG132 on DN. This inconsistence may be due to the discrepancy between in vivo and in vitro study. On one hand, exposure of renal tubule cells to high-glucose cannot fully mimic diabetes-induced renal injury. On the other hand, kidney is composed of several different kinds of cells, such as mesangial cells, tubular epithelial cells, endothelial cells, fibroblasts, and podocytes, not just tubule cells.

**Figure 7:**
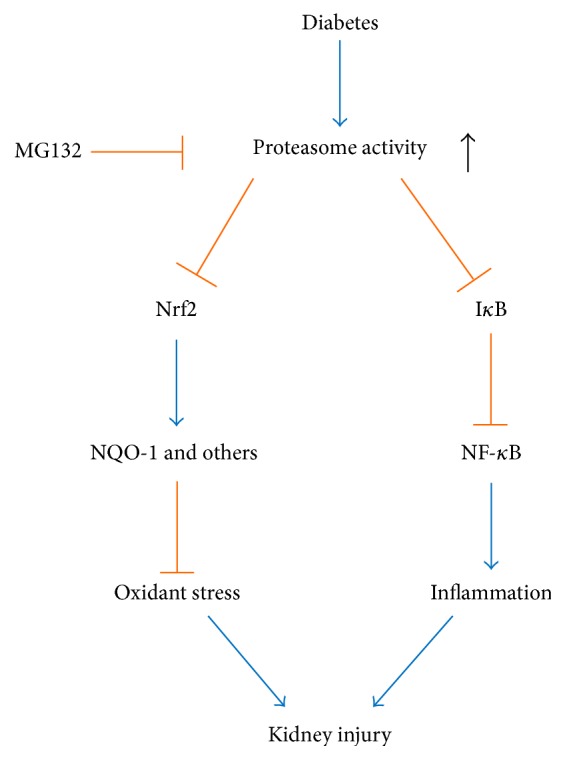
Sketch map of possible mechanisms under which MG132 attenuates DN. MG132 inhibits proteasome activity, leading to the upregulation of Nrf2 and I*κ*B. For one thing, antioxidant genes, such as* NQO-1*, are activated due to the upregulation of Nrf2; therefore, renal oxidative damage is reduced. On the other hand, NF-*κ*B is downregulated secondary to the upregulation of I*κ*B, resulting in the reduction of inflammation. Consequently, MG132 alleviates diabetic nephropathy.

Several studies showed that diabetes increased proteasomal activity in vein endothelial cells, heart, and gastrocnemius muscles [28–32]. The present study also demonstrated diabetes increased renal proteasomal activity, companied by the decrease in I*κ*B and increase in NF-*κ*B. MG132 treatment significantly inhibited proteasomal activity, companied by the upregulation of I*κ*B and downregulation of NF-*κ*B. It is widely accepted that inflammation contributes to the pathogenesis and progression of DN. NF-*κ*B is a protein complex that controls many genes involved in inflammation. Several studies showed that MG132 inhibited the expression of inflammatory cytokines [33, 34], but its mechanism was not fully known. Under physiological conditions, NF-*κ*B is sequestered in the cytoplasm by its inhibitor, called I*κ*B [35–37]. When activated by signals, I*κ*B is phosphorylated and ubiquitinated, which then leads them to be degraded by the proteasome [38, 39]. With the degradation of I*κ*B, NF-*κ*B complex is freed to enter the nucleus where it promotes the expression of specific genes that encodes proinflammatory cytokines. In the present study, MG132, the proteasome inhibitor, increased I*κ*B and decreased NF-*κ*B. Thus, it is reasonable to assume that MG132 inhibited proteasomal activity, resulting in the decrease in I*κ*B degradation. Consequently, NF-*κ*B was retained in the cytoplasm, which prevented NF-*κ*B activation. Finally, MG132 suppressed renal inflammation. The possible mechanisms by which MG132 ameliorated DN were shown in Figure 7.

Current study showed that MG132 decreased TGF-*β* expression in diabetic mice. It is widely accepted that both oxidative stress and inflammation lead to TGF-*β* activation and fibrosis. In addition, in current study, we hypothesized that MG132 suppressed both oxidative stress via Nrf2 upregulation and inflammation via NF-*κ*B downregulation (Figure 7). Therefore, MG132 mediated oxidative stress and inflammation inhibition may explain the phenomenon of TGF-*β* reduction. In a recent study, Huang et al. suggested that MG132 alleviated DN by inhibiting TGF-*β* signaling and this effect was associated with the ability of MG132 to reduce the degradation of SnoN protein [40]. This might be another mechanism by which MG132 decreased TGF-*β*.

In summary, the present study demonstrates for the first time that proteasome inhibitor MG132 at low dose ameliorates DN by both induction of Nrf2 and inhibiting NF-*κ*B via upregulation of I*κ*B. As we reported before [6], the dosage of MG132 in current study (10 *μ*g/kg/day) was the lowest dosage reported in the literature in vivo. Since proteasome activity increased in diabetic kidney, whether increasing MG132 dosage (nontoxic) can enhance its effectiveness needs to be further investigated in DN models.

## Figures and Tables

**Table 1:** General changes after streptozotocin injection for 5 consecutive days.

	Control	MG132	DM	DM/MG132
UACR				
Wild type	25.21 ± 3.12	24.81 ± 3.12	28.65 ± 5.64	29.23 ± 5.87
Nrf2-KO	27.81 ± 3.76	27.32 ± 3.89	31.23 ± 5.92	30.89 ± 6.03
Blood glucose				
Wild type	98.89 ± 10.32	100.65 ± 11.20	280.46 ± 32.23^*∗*^	290.85 ± 35.03^*∗*^
Nrf2-KO	100.74 ± 12.32	96.15 ± 10.86	295.21 ± 35.23^*∗*^	289.75 ± 34.98^*∗*^
Body weight				
Wild type	19.65 ± 2.06	19.52 ± 2.16	19.32 ± 1.99	19.61 ± 2.03
Nrf2-KO	18.96 ± 1.96	19.32 ± 2.12	19.45 ± 2.15	19.1 ± 2.2

*Notes*. Data are presented as mean ± SD. ^*∗*^*p* < 0.05 versus wild type/control or Nrf2-KO/control correspondingly.
